# A novel fully human anti-NCL immunoRNase for triple-negative breast cancer therapy

**DOI:** 10.18632/oncotarget.13522

**Published:** 2016-11-23

**Authors:** Chiara D'Avino, Dario Palmieri, Ashley Braddom, Nicola Zanesi, Cindy James, Sara Cole, Francesco Salvatore, Carlo M. Croce, Claudia De Lorenzo

**Affiliations:** ^1^ Department of Molecular Medicine and Medical Biotechnology, University of Naples “Federico II”, 80131 Naples, Italy; ^2^ Ceinge Advanced Biotechnology S.C.ar.l., 80145 Naples, Italy; ^3^ Department of Molecular Virology, Immunology and Medical Genetics, The Ohio State University, Columbus, 43210 Ohio, USA; ^4^ Department of Mass Spectroscopy and Proteomics, The Ohio State University, Columbus, 43210 Ohio, USA; ^5^ Campus Microscopy and Imaging Facility, The Ohio State University, Columbus, 43210 Ohio, USA

**Keywords:** triple negative breast cancer, cancer immunotherapy, nucleolin, human RNase, microRNA

## Abstract

Breast cancer is the most common cancer in women worldwide. A new promising anti-cancer therapy involves the use of monoclonal antibodies specific for target tumor-associated antigens (TAAs). A TAA of interest for immunotherapy of Triple Negative Breast Cancer (TNBC) is nucleolin (NCL), a multifunctional protein, selectively expressed on the surface of cancer cells, which regulates the biogenesis of specific microRNAs (miRNAs) involved in tumor development and drug-resistance. We previously isolated a novel human anti-NCL scFv, called 4LB5, that is endowed with selective anti-tumor effects. Here we report the construction and characterization of a novel immunoRNase constituted by 4LB5 and a human pancreatic RNase (HP-RNase) called “4LB5-HP-RNase”. This immunoRNase retains both the enzymatic activity of human pancreatic RNase and the specific binding of the parental scFv to a panel of surface NCL-positive breast cancer cells. Notably, 4LB5-HP-RNase dramatically and selectively reduced the viability and proliferation of NCL-positive tumor cells *in vitro* and *in vivo*. Specifically, it induced apoptosis and reduced the levels of the tumorigenic miRNAs miR-21, -221 and -222. Thus, this novel immunoagent could be a valuable tool for the treatment of TNBC patients ineligible for currently available targeted treatments.

## INTRODUCTION

Breast cancer (BC) will affect one in eight (12%) women during their lives and almost half a million patients lose their life to BC annually, thus being the most frequent cancer among women [1]. Trastuzumab (Herceptin), a humanized antibody, is in clinical use for ErbB2-positive breast carcinoma [2, 3]. However, ErbB2 overexpression/amplification occurs in only ∼25% of BC patients [4] many of whom are *de novo* resistant or will acquire resistance to this treatment [5]. Furthermore, concerns have been raised about the cardiotoxicity of Trastuzumab and other anti-ErbB2 drugs [6]. Consequently, there is a need for new specific targets for the therapy of anti-ErbB2-resistant breast cancer, including Triple Negative Breast Cancer (TNBC), which lacks estrogen receptor (ER), progesterone receptor and ErbB2. TNBC accounts for ∼14% of all breast cancers and about 170,000 new TNBC diagnoses per year [7]. These patients develop a malignant phenotype, and their death rate is higher than any other type of BC (median overall survival around 12 months in the metastatic setting) [8].

A new attractive target for TNBC immunotherapy is nucleolin (NCL), a major nucleolar protein [9] that is directly involved in ribosomal processing [10, 11]. Nucleolin is a multi-functional protein that is involved in the metabolism of nucleic acids (DNA and RNA), rRNA maturation, and ribosome biogenesis. Nucleolin is mainly localized in the nucleus, but is also overexpressed in the cytoplasm and on the surface of leukemic, renal, pulmonary, prostate, intestinal, breast, hepatic, kidney, cervical, colon cancer cells, melanomas and gliomas [10, 12–14]. Altered NCL surface expression and localization is directly or indirectly involved in signal transduction events subsequent to its interaction with several molecules/receptors on the cell surface that are involved in cell growth, tumor invasiveness, inflammation and/or angiogenesis. Thus, surface NCL is an attractive target for customized breast cancer therapy also because it is continuously overexpressed on the tumor cell surface and is associated with malignant proliferation irrespective of nuclear NCL [15].

Consistent with its involvement in RNA processing, we previously reported that NCL promotes the maturation of a specific set of microRNAs (miRNAs), namely, miR-21, miR-221 and miR-222, whose up-regulation is involved in breast tumorigenesis, metastasis formation, and drug resistance [16–19]. Accordingly, NCL inhibition resulted in a decrease in the oncogenic potential of TNBC cells *in vitro* and *in vivo*. This finding confirms the importance of tumorigenic miRNAs in immune escape, neovascularization and metastasis, as well as the key role played by NCL in affecting their levels [11].

Aptamers (AS1411) [20] and peptides [21–22], specific for NCL, are available that bind and inhibit this protein in cancer cells. These compounds have been proposed as vehicles for the specific delivery of antitumor moieties into cancer cells [23]. Although promising, NCL-targeting aptamers and peptides could have an extremely short *in vivo* half-life due to their small molecular size and thus fail in *in vivo* therapies.

To circumvent these problems, we previously isolated a novel fully human anti-NCL scFv, called “4LB5”, by phage-display technology [24]. This immunoagent recognizes the RNA binding domain of NCL, selectively binds with high affinity to it on the cell membrane, and discriminates between breast tumor and normal cells. 4LB5 is also internalized in the cytoplasm of target cells, reduces their cell viability and growth both in cell cultures and in animal models, and abrogates the biogenesis of NCL-dependent miRNAs [24]. In the attempt to enhance the potential therapeutic effect of 4LB5, we have engineered a novel anti-NCL fully human immunoRNase (IR), called “4LB5-HP-RNase”, by fusing 4LB5 with the human pancreatic ribonuclease in a similar manner to the previously described anti-ErbB2 IR [25].

Here we describe the production and characterization of this novel IR and report its antitumor effects both *in vitro* and *in vivo*.

## RESULTS

### Construction, expression and purification of the novel anti-Nucleolin immunoRNase 4LB5-HP-RNase

**Figure 1:**
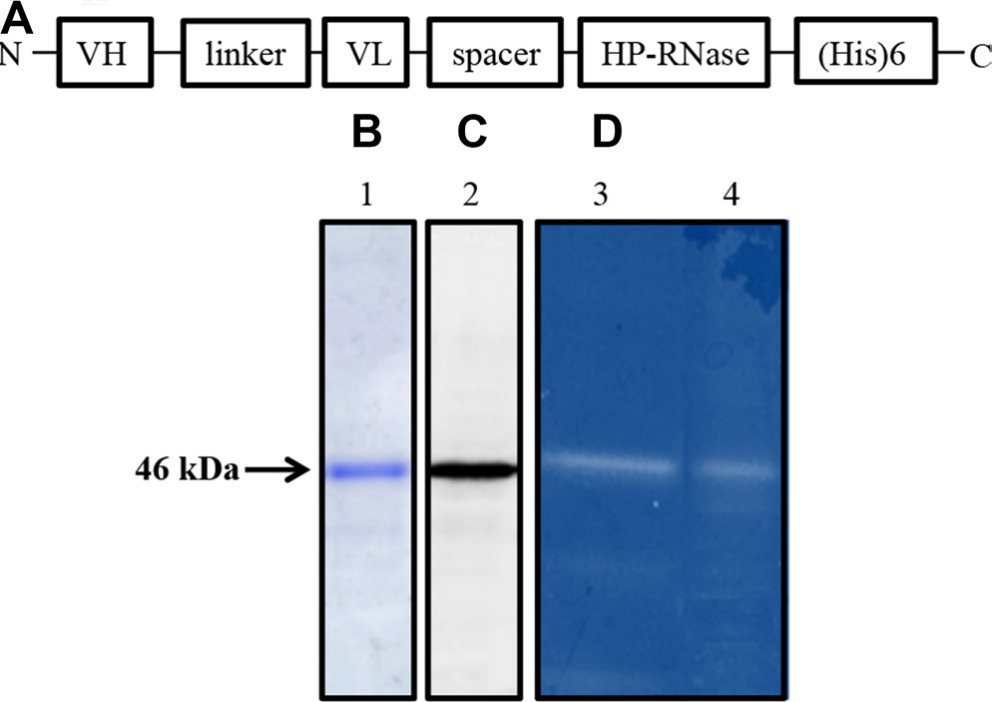
Schematic representation and biochemical analyses of purified 4LB5-HP-RNase (**A**) Schematic representation of the human anti-Nucleolin immunoRNase 4LB5-HP-RNase. VH and VL, the Variable Heavy and Light chains of the anti-Nucleolin scFv; linker, the flexible oligopeptide conjugating VH and VL; spacer, the peptide connecting the scFv and RNase; HP-RNase, human pancreatic RNase. (**B**) SDS-PAGE analysis followed by Coomassie staining of the sample eluted by IMAC. *Lane 1*, fraction eluted from the column. (**C**) Western blotting analysis using an anti-His antibody of the sample as in *Lane 1*. (**D**) Zymogram of 4LB5-HP-RNase (*Lane 4*) using yeast RNA as a substrate; *Lane 3*, enzymatic activity of ERB-HP-RNase used as positive control of the native human pancreatic ribonuclease activity.

The chimeric construct encoding 4LB5-HP-RNase was generated by genetically fusing the cDNAs encoding the human pancreatic RNase (HP-RNase) and the anti-NCL scFv 4LB5, as described under Materials and Methods. The resulting construct has the scFv at the NH_2_- end followed by a spacer included between the antibody moiety and the RNase, which is at the C-terminal end, followed by a hexahistidine tag for the detection and purification of the chimeric construct (Figure 1A). The recombinant 4LB5-HP-RNase was expressed in *Escherichia coli*, purified by affinity chromatography on a resin with immobilized cobalt ions, as described under Materials and Methods, and analyzed by SDS-PAGE. As shown in Figure 1B, a single product with a correct molecular weight of about 46 kDa was detected, thus indicating that the recombinant protein was homogeneous. Western blotting analyses with an anti-Histidine antibody revealed a product with the expected molecular weight, that corresponded to the fusion protein (Figure 1C). The fusion protein was then tested for its enzymatic activity by a zymogram, developed by using yeast RNA as a substrate, as described in “Materials and Methods”. As illustrated in Figure 1D, a single active band was detected that corresponded to the molecular weight of 4LB5-HP-RNase, thus indicating that the immunoRNase is endowed with RNase activity of native human pancreatic ribonuclease comparable to that of another immunoRNase, ERB-HP-RNase, previously described [25], used as positive control.

### 4LB5-HP-RNase binds to NCL on the membrane of tumor cells

**Figure 2:**
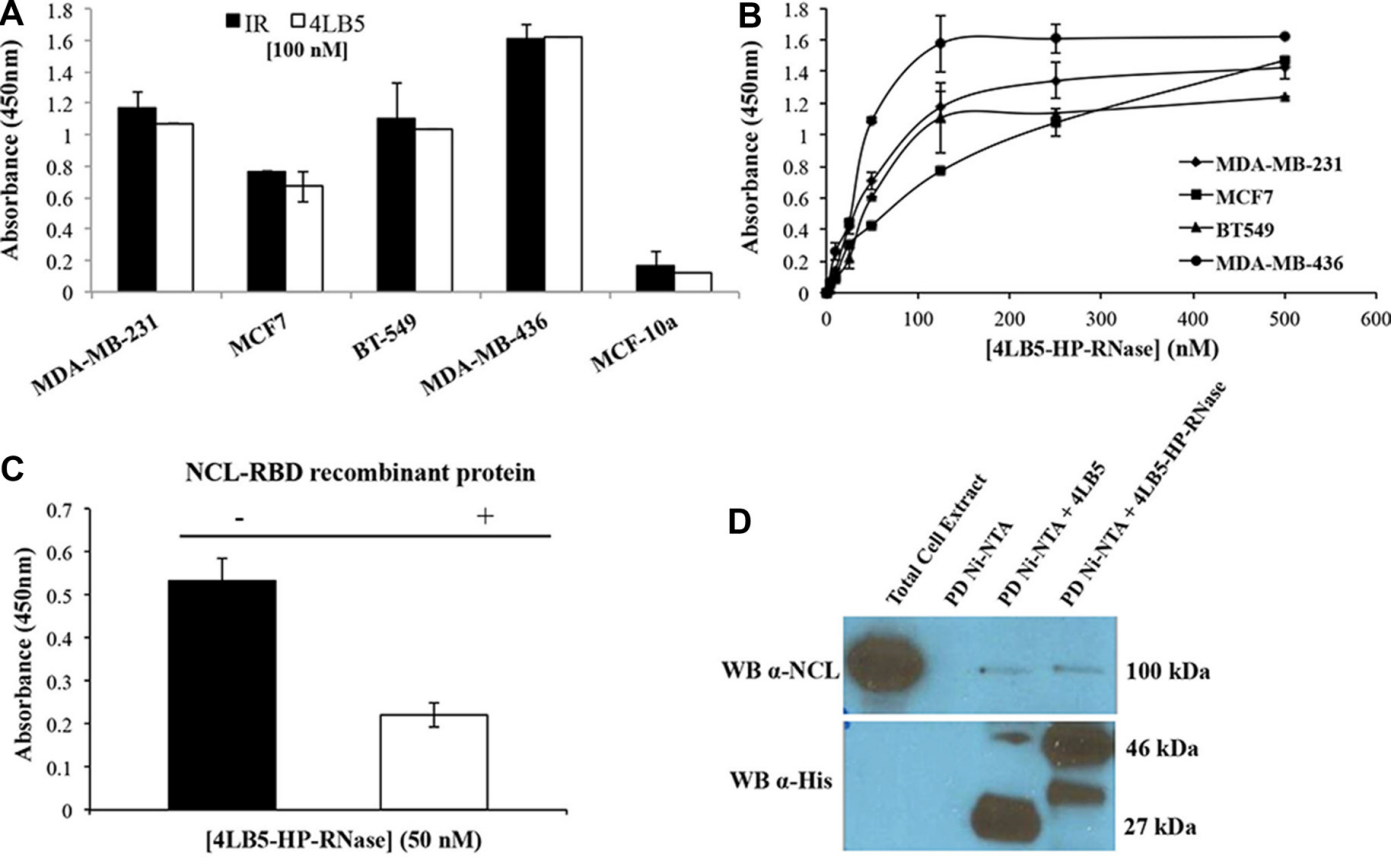
Binding of 4LB5-HP-RNase to NCL on cancer cells (**A**) The binding of 4LB5-HP-RNase to NCL-positive MDA-MB-231, MCF-7, BT-549 or MDA-MB-436 breast cancer cells or to NCL-negative MCF-10a normal-like breast cells was tested by incubating the chimeric protein (100 nM) with the cells; 4LB5 was tested at equimolar doses as a positive control in parallel assays. (**B**) Binding curves of 4LB5-HP-RNase to surface NCL-positive MDA-MB-231 (rhomboids), MCF-7 (squares), BT-549 (triangles), MDA-MB-436 (circles) breast cancer cell lines performed by using increasing concentrations (5–500 nM) of the immunoRNase. All the experiments are representative of three independent experiments performed in triplicate. Mean + SD is reported. (**C**) ELISA assay performed by testing 4LB5-HP-RNase (50 nM) on MDA-MB-231 breast cancer cells in the absence (black) or in the presence (white) of equimolar doses of NCL-RBD recombinant protein. All the experiments are representative of three independent experiments performed in triplicate. Mean + SD is reported. (**D**) Pull-down assay performed on lysates of MDA-MB-231 cells using Ni-NTA resin and 4LB5 or 4LB5-HP-RNase. Ni-NTA resin incubated with total cell lysate in the absence of 4LB5 or 4LB5-HP-RNase was used as negative control. Anti-NCL antibody (upper panel) and anti-His antibody (Lower panel) were used to visualize the effective pull-down of endogenous NCL in the presence of either 4LB5 or 4LB5-HP-RNase.

Although NCL is predominantly localized in the nucleolus, NCL is overexpressed in the cytoplasm and on the membrane of many different tumor cell types [10]. Accordingly, we demonstrated that anti-NCL scFv 4LB5 specifically binds to NCL on the surface of actively proliferating breast cancer cells [24]. To evaluate the ability of the chimeric protein 4LB5-HP-RNase to bind to NCL-positive cells, we carried out an ELISA assay on a panel of breast tumor cells, such as MDA-MB-231, MDA-MB-436, BT-549 (all Triple-Negative Breast Cancer cells) and MCF7 cells (luminal epithelial breast cancer), expressing high levels of surface-NCL. In parallel assays, we used normal-like MCF10a breast cells that express low levels of surface-NCL as a negative control. As shown in Figure 2A, 4LB5-HP-RNase specifically binds to all NCL-positive cancer cell lines with an affinity similar to that of the parental 4LB5 scFv, tested in a parallel assay, but not to MCF-10a normal-like mammary cells.

To evaluate the binding affinity of 4LB5-HP-RNase to NCL expressed on live cells, we constructed binding curves on each cell line treated with increasing concentrations (5–500 nM) of the purified immunoRNase, as described in “Materials and Methods”. As shown in Figure 2B, 4LB5-HP-RNase efficiently binds to the surface of these cells with an apparent affinity in the nanomolar range (5–50 nM). To determine whether the detected binding was due to the specific interaction between 4LB5 and NCL, we analyzed the binding of 4LB5-HP-RNase to MDA-MB-231 cells by ELISA assay before and after its pre-incubation with equimolar doses of NCL-RNA Binding Domain (RBD) recombinant protein used as a bait for the initial identification of 4LB5 scFv [24]. As shown in Figure 2C, 4LB5-HP-RNase binding to cells was significantly lower (over 50%) in the presence of NCL-RBD than in its absence.

Finally, we performed a pull-down assay to verify the interaction between endogenous NCL and 4LB5-HP-RNase. To this end, we incubated total cell extracts of MDA-MB-231 cells with either 4LB5-HP-RNase or 4LB5 and loaded them on a high-capacity Ni-NTA resin to isolate the protein complexes by His-tag binding of the chimeric construct (see Materials and Methods). Bound NCL trapped in immunocomplexes with 4LB5-HP-RNase or 4LB5 was detected by immunoblotting using a commercially available anti-NCL mAb as primary antibody. As shown in Figure 2D, immunoblotting with an anti-His antibody confirmed similar pull-down of the 4LB5-HP-RNase (46 kDa) and 4LB5 (27 kDa) proteins. Immunoreactive bands of the expected molecular size corresponding to NCL (100 kDa) were observed in total cell extracts after NCL pull-down with either 4LB5-HP-RNase or 4LB5 with comparable intensity, thus confirming that 4LB5-HP-RNase specifically binds to NCL in a manner similar to that of the parental 4LB5 scFv. In the sample of cell extracts treated with 4LB5-HP-RNase is visible an additional band of lower molecular weight that could be a product derived from degradation of the immunoRNase likely in the spacer between the scFv and RNase.

### 4LB5-HP-RNase is taken up by breast tumor cells

**Figure 3:**
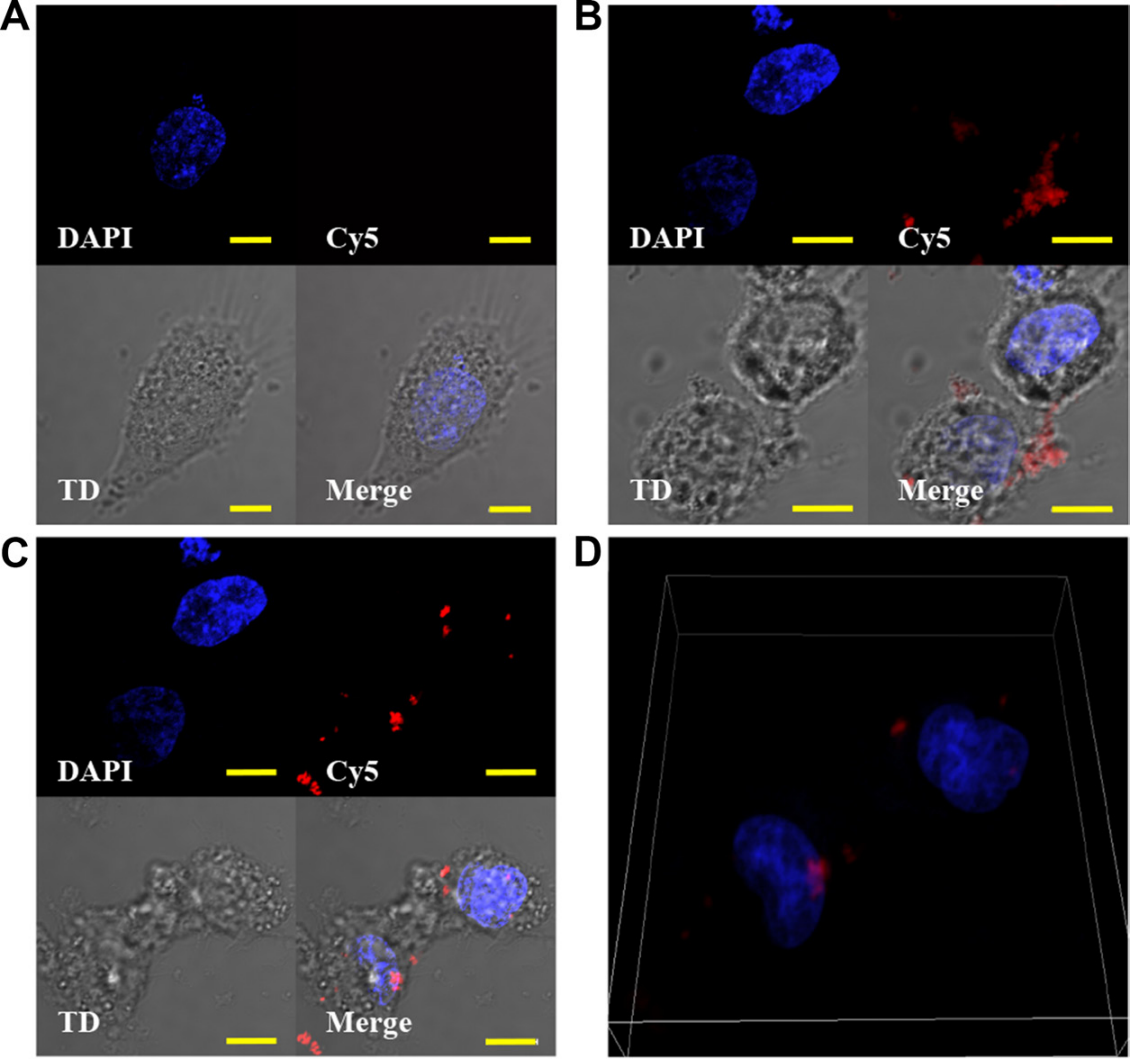
4LB5-HP-RNase is internalized by TNBC cells (**A**, **C**) MDA-MB-231 cells were cultured in the presence of Cy5 (A), 4LB5-Cy5 (**B**) or 4LB5-HP-RNase-Cy5 (C) for 4 h at 37°C. Labeled compounds that were not cell-associated were washed away. Cells were incubated with Hoechst nuclear staining, and microscopy images were acquired. For each experimental condition, Hoechst, Cy5 image stacks are presented as a 2 dimensional projection along with a single representative DIC image (TD). A merged image is also presented for each treatment. (**D**) 3D-rendering of the image series through the volume of 4LB5-HP-RNase (from 3C) treated cells to help visualization of the intracellular localization of 4LB5-HP-RNase. Scale bar = 10 μm.

**Figure 4:**
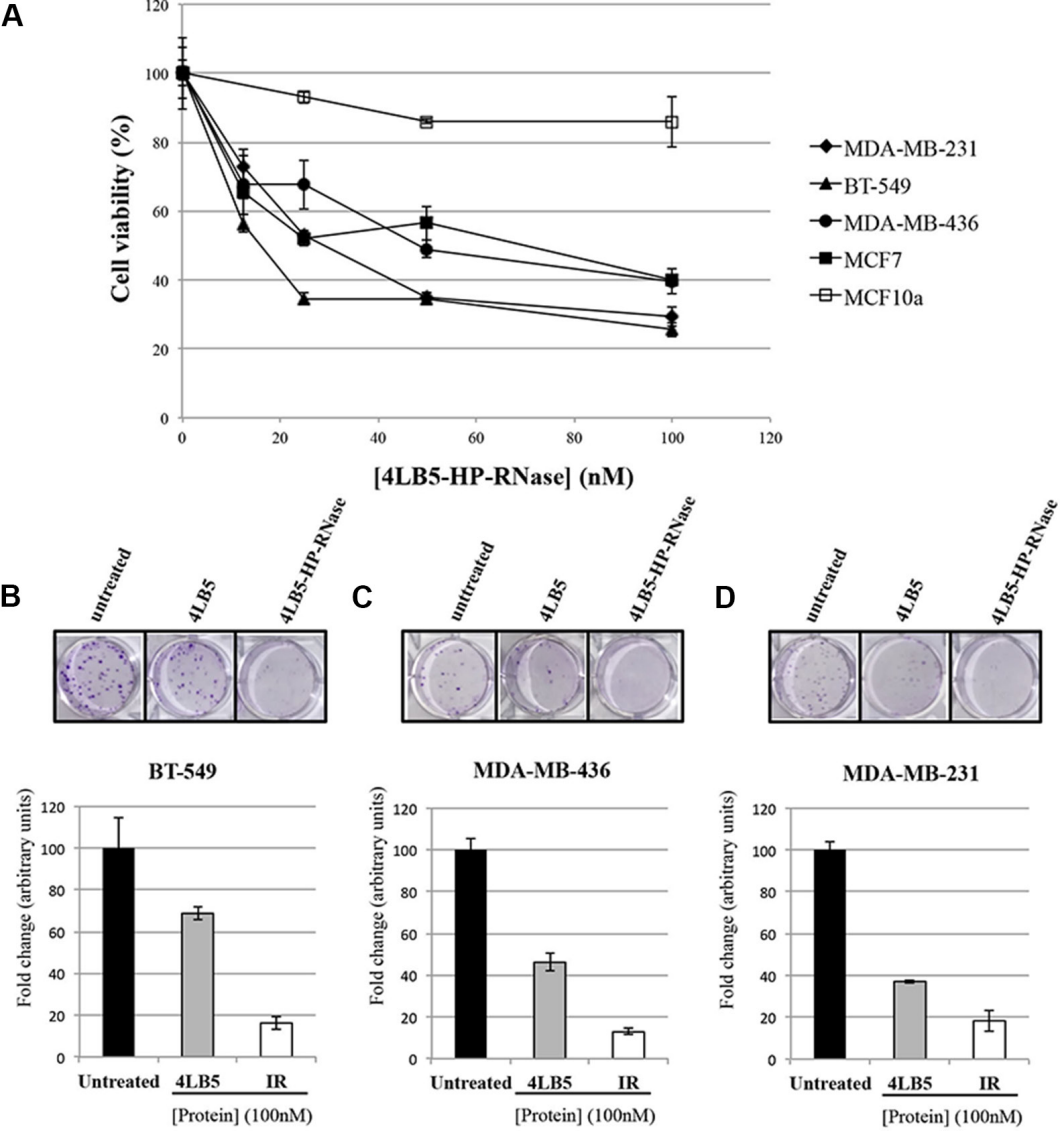
*In vitro* effects of the immunoRNase on cell proliferation (**A**) Dose-response curves of NCL-positive MDA-MB-231 (rhomboids), BT-549 (triangles), MDA-MB-436 (circles) and MCF-7 (black squares) breast cancer cells or NCL-negative MCF10a (empty squares) normal-like breast cells treated for 72 h with increasing doses (10–100 nM) of 4LB5-HP-RNase. Colony assays on BT-549 (**B**) MDA-MB-436 (**C**) and MDA-MB-231 (**D**) cells performed in the absence or in the presence of 100 nM of 4LB5-HP-RNase or 4LB5, tested in parallel assays, and stained after 10 days with Crystal violet. All the experiments are representative of three independent experiments performed in triplicate.

We previously reported that, after interaction with NCL, 4LB5 is actively internalized into breast cancer cells [24]. To determine if also its derived immunoRNase is internalized by TNBC cells, 4LB5-HP-RNase was *in vitro* labeled with Cy5 fluorescent dye and added to culture medium of MDA-MB-231 cells for 4 h at 37°C. As control, cells were also incubated with 4LB5-Cy5 and with Cy5 alone, for background evaluation. A series of images through the full volume of the cells (z-stacks) was analyzed by using confocal microscopy. As expected, no Cy5 fluorescent signal was observed when cells were treated with unconjugated fluorescent dye (Figure 3A). Intracellular localization was observed for both 4LB5-Cy5 (Figure 3B) and 4LB5-HP-RNase-Cy5 (Figure 3C). While much of the signal was intracellular, some extracellular 4LB5-HP-RNase and 4LB5 was also observed. We performed analysis of the Mean Fluorescent Intensity (MFI) of intracellular and extracellular Cy5 signal for Figure 3B and 3C and determined that the ratio between intracellular and extracellular signal was 3.86 and 1.72 for 4LB5-HP-RNase and 4LB5, respectively. These data demonstrate that the majority of 4LB5-HP-RNase is being internalized. A three dimensional rendering of the data shown in Figure 3C presented in Figure 3D and a Supplementary Video S1 further demonstrate that the 4LB5-HP-RNase-Cy5 is taken up by the cells. Collectively, these data are consistent with previous results showing that 4LB5 is internalized [24] by NCL-positive tumor cells.

### 4LB5-HP-RNase inhibits tumor cell growth *in vitro*

NCL inhibition induced with anti-NCL aptamer AS1411 [11] or scFv 4LB5 [24] impairs breast cancer cell proliferation *in vitro* and *in vivo*. Thus, we hypothesized that the fusion compound could exert enhanced cytotoxic effects, thereby combining the anti-neoplastic properties of both 4LB5 and HP-RNase, following intra-cytoplasmic delivery. To address this hypothesis, we first evaluated the ability of 4LB5– HP- RNase to exert a specific cytotoxic effect on NCL-positive cells by incubating MDA-MB-231, MDA-MB-436, BT-549 or MCF7 breast cancer cells with and without increasing concentrations of the protein (10–200 nM). After treatment for 72 h at 37°C, cell survival was analyzed by either trypan blue-exclusion cell counting by using an Automated Cell Counter TC10 (Bio-Rad, Richmond, CA, USA) or by MTT assays. As shown in Figure 4A, 4LB5–HP-RNase selectively and dose-dependently inhibited the viability of the NCL-positive MDA-MB-231, MDA-MB-436, BT-549 and MCF7 cells, with an IC_50_ of about 25 nM, 50 nM, 12,5 nM and 25 nM, respectively. These results were confirmed also by MTT assays. 4LB5–HP-RNase exerted only slight effects on NCL-negative MCF-10a (normal-like) breast control cells and only at high concentrations.

To verify the antitumor effects of the new construct and to compare them to those of 4LB5, we tested the effects of 4LB5-HP-RNase on the proliferation of surface-NCL-positive MDA-MB-231, MDA-MB-436 and BT-549 breast cancer cells in colony formation assays.

The results indicate that the number of colonies in the sample treated with 4LB5-HP-RNase is much lower than that of negative control cells and significantly reduced with respect to cells treated with the parental 4LB5 scFv tested in parallel assays (Figure 4B–4D), which indicates that, probably due to its acquired RNase activity, 4LB5-HP-RNase inhibits the proliferation of all treated cells more efficiently than do the parental scFv.

### 4LB5-HP-RNase induces apoptosis in surface NCL-positive cancer cells

**Figure 5:**
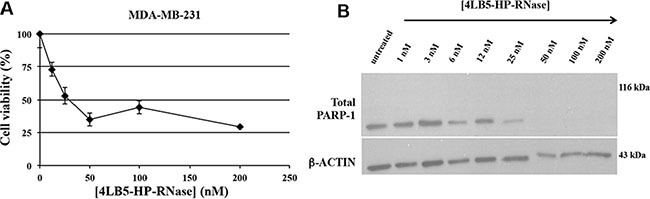
Effects of 4LB5-HP-RNase on cancer cell apoptosis (**A**) Dose-response curve of NCL-positive MDA-MB-231 cells (rhomboids) treated for 72 h with increasing doses (10–200 nM) of 4LB5-HP-RNase. (**B**) Western Blotting analysis of total lysates from MDA-MB-231 cells treated with increasing concentrations (10–200 nM) of 4LB5-HP-RNase to evaluate PARP cleavage as a marker of apoptosis activation. Actin was used as loading control.

Several recent reports indicate that NCL negatively regulates cell apoptosis [11]. The role of cell surface NCL is highlighted by studies showing that functional blockage or down-regulation of cell surface NCL expression results in migration inhibition and tube formation [26, 27], and causes endothelial-cell apoptosis [28]. To investigate whether the reduction of the viability and proliferation of surface-NCL cells treated with 4LB5-HP-RNase is associated with its ability to induce apoptosis, the MDA-MB-231 TNBC cells were incubated with increasing amounts of 4LB5-HP-RNase (1–200 nM). After 72 hours of exposure to 4LB5-HP-RNase, we measured the levels of not cleaved poly(ADP-ribose) polymerase 1 (PARP-1), which is degraded during apoptosis [29], in total cell extracts. As shown in Figure 5A and 5B, 4LB5-HP-RNase dose-dependently degraded PARP; in fact, no PARP signal was observed after treatment with 50 nM protein, which confirms that anti-NCL immunoRNase activates the apoptotic pathway.

### Effects of 4LB5-HP-RNase on the levels of oncogenic intracellular and extracellular miRNAs

**Figure 6:**
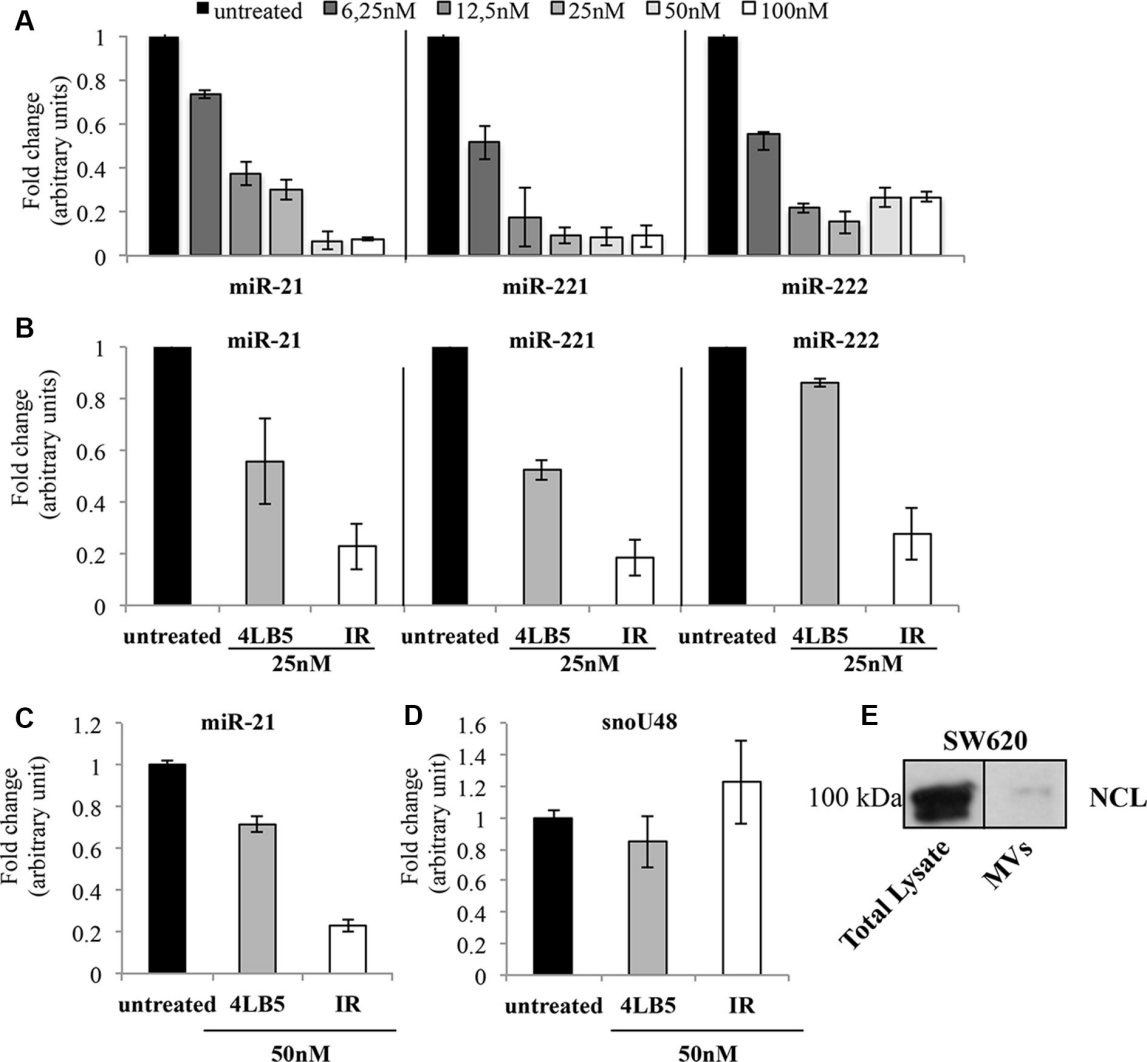
Effects of 4LB5-HP-RNase on cellular and EV-associated miRNAs (**A**) NCL-dependent mature miR-21, -221 and -222 levels were analyzed by Real-Time PCR after 72 h of incubation of MDA-MB-231 cells with increasing concentrations (6–100 nM) of 4LB5-HP-RNase. (**B**) Comparison between the effects of 4LB5 (grey bars) and 4LB5-HP-RNase (white bars) on mature miR-21, -221 and -222 levels by using an equimolar concentration (25 nM) of compounds for the treatment of MDA-MB-231 cells. Relative microRNA expression levels were normalized for control untreated sample, and relative abundance (fold change) is reported. Data are the average of three independent experiments performed in triplicate. Mean + SD is reported. (**C**) The mature miR-21 levels were analyzed by Real-Time PCR on RNA extracted from exosomes derived from the conditioned medium of SW620 cancer cells treated for 72 h with equimolar doses (50 nM) of 4LB5-HP-RNase (white bars) or 4LB5 (grey bars), tested in a parallel assay. Relative microRNA expression levels were normalized for control untreated sample, and relative abundance (fold change) is reported. (**D**) The snoRNA RNU48 was used as negative control of RNAs cellular contamination. (**E**) Western Blotting analysis showing NCL in both total lysates and MVs from SW620 cells, used for the experiments reported in C and D.

The critical role of NCL in microRNA biology was recently revealed by the finding that NCL regulates the maturation and expression of specific miRNAs, including *miR-21, miR-221, miR-222*, that are involved in the diverse phases of breast cancer development, and drug resistance [11, 30–33]. To assess if the chimeric 4LB5-HP-RNase protein affects the levels of these miRNAs, triple-negative MDA-MB-231 breast tumor cells were incubated with increasing doses of 4LB5-HP-RNase, and the RNA was extracted after 72 hours of treatment. As shown in Figure 6A, Real-Time PCR analysis revealed that 4LB5-HP-RNase dose-dependently reduced the levels of the mature miR-21, miR-221 and miR-222 with a relevant effect already at the lowest concentration tested. A previous study demonstrated that 4LB5 alone inhibited the interaction between NCL and the micro-RNA microprocessor complex thereby impairing the maturation of these NCL-dependent miRNAs [24].

To evaluate if the effects exerted by 4LB5-HP-RNase on mature miRNAs levels were due also to RNA degradation of the ribonuclease moiety and not only to inhibition, mediated by the scFv domain, of the interaction of NCL with the microprocessor complex, MDA-MB-231 cells were incubated in parallel assays with equimolar doses of 4LB5 or 4LB5-HP-RNase. As shown in Figure 6B, the immunoRNase downregulates miRNAs levels more efficiently than does 4LB5, which suggests that the enzymatic activity of the ribonuclease greatly enhances the effect of 4LB5 on miRNAs levels. These results demonstrate that 4LB5-HP-RNase efficiently impairs the levels of miRNAs involved in malignancy of mammary cancer cells resistant to conventional anti-cancer therapies, and that this effect is due also to its enzymatic activity.

The release of extracellular vesicles (EVs) from tumor cells is increasingly attracting attention due to its role in cancer intercellular communication. A key component of the EV-associated cargo are miRNAs, which can be delivered to surrounding cells, and thus exert their oncogenic role [34–36]. Since cancer cells release EVs into surrounding tissues, which play pleiotropic roles in cancer progression, namely, immune escape, neovascularization and metastasis, we tested the effects of 4LB5-HP-RNase on EV-packed miRNAs. Notably, oncogenic miR-21, miR-221 and miR-222 were previously demonstrated to be released by colon cancer cells and transferred to other cancer cells, thereby mediating their aggressiveness [37].

To address the hypothesis that NCL protects cancer cell-derived EV-associated miRNAs, we evaluated the ability of anti-NCL 4LB5-HP-RNase and 4LB5 scFv to modify the levels of EV-related miR-21 after the binding and the possible displacement of microRNAs from NCL. To this end, we first isolated EVs from the conditioned serum-deprived medium of lymph node metastatic derivatives of colorectal SW620 cancer cells that are widely used as a model for exosome studies [38]. Then, using a combination of a polymer-based exosome precipitation and a differential centrifugation approach to fractionate the EV-containing medium, we isolated the SW620-derived exosomes and tested them for the presence of NCL by Western blotting (Figure 6E). Once confirmed the presence of NCL, EVs were treated with 4LB5-HP-RNase or 4LB5 scFv at 50 nM for 2 hours at 37°C. Total RNA was extracted and exosome-related miR-21 levels were analyzed by Real-Time PCR analysis. RNU48 cellular snoRNA, served as a negative control, to evidence the potential contamination of cellular RNAs during the EV purification procedure Figure 6D. As shown in Figure 6C, miR-21 levels were much lower in 4LB5-HP-RNase-treated cells than in the untreated control, whereas treatment with an equimolar dose of 4LB5, tested in a parallel assay, only slightly affected miR-21 levels. These data support the hypothesis that the binding of NCL by the scFv 4LB5 may cause displacement of miRNAs from NCL thus enabling the RNase moiety present in 4LB5-HP-RNase to degrade miR-21. Thus, the enzymatic activity of 4LB5-HP-RNase could be responsible for its more potent antitumor effects on extracellular miRNAs, which indicates that the anti-NCL immunoRNase may play a role in the inhibition of the inter-cellular communication involved in tumor progression.

### *In vivo* effects of 4LB5-HP-RNase

**Figure 7:**
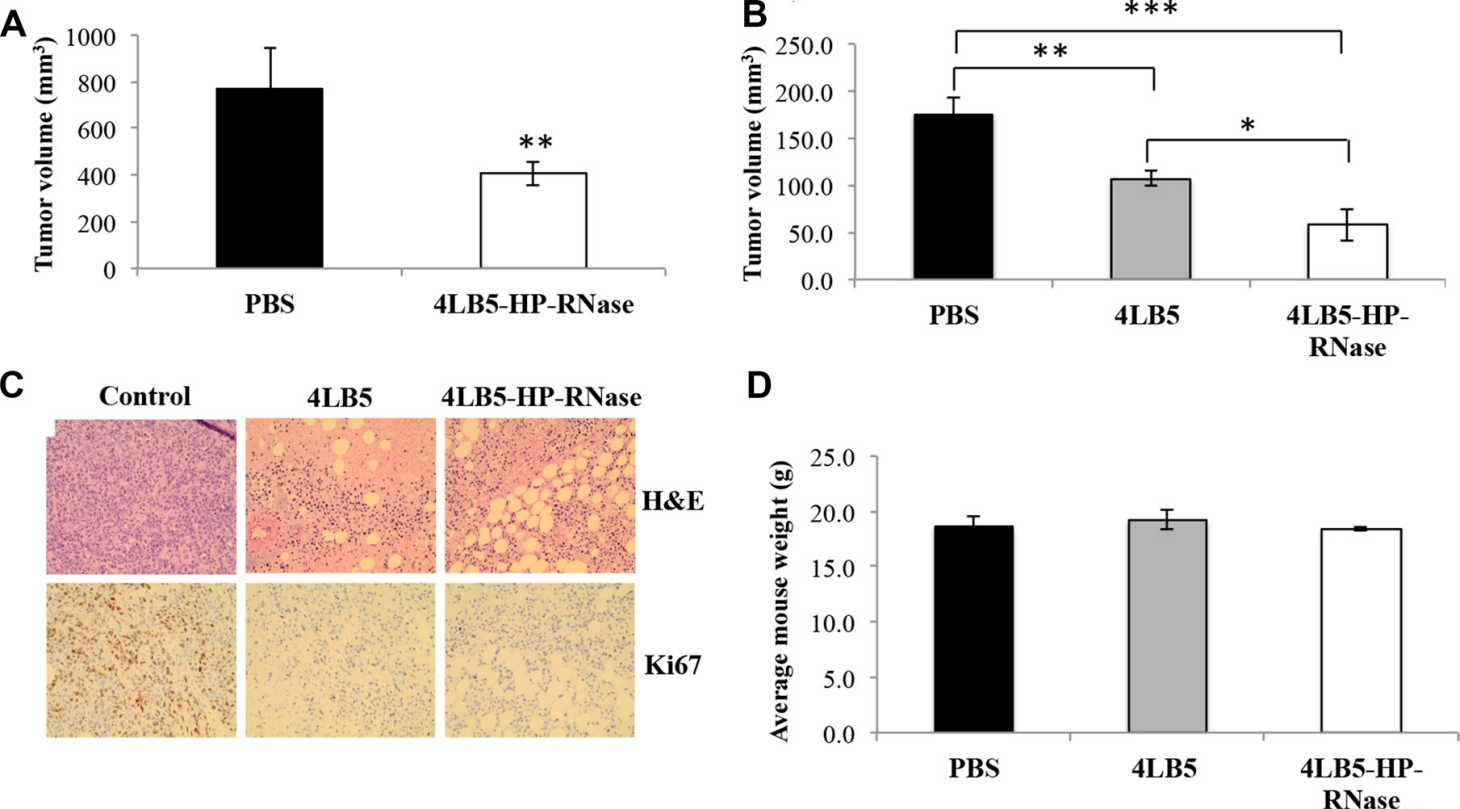
*In vivo* effects of 4LB5-HP-RNase on NCL-positive tumors induced in mice by using MDA-MB-231 breast cancer cells (**A**) NOD-SCID (*n* = 5) mice were injected with 2 × 10^6^ MDA-MB-231-Luc cells into the mammary fat pad. After 2 weeks, mice were treated with control PBS solution or 2 mg/kg of 4LB5-HP-RNase twice a week. Mice were monitored by IVIS weekly. At 6 week from injection (4 weeks of treatment), mice were sacrificed. Tumors were excised and measured. **P < 0.01 (**B**) In a second experiment, mice were treated with equimolar doses of 4LB5 or 4LB5-HP-RNase (2 mg/kg) or with PBS, as described for Figure 7A. *P < 0.05 (**C**) Representative images of H&E and Ki-67 staining of tumor tissue slices obtained from different fields are shown; 20× magnification is reported. (**D**) Body weight of mice treated with PBS (black bars), 4LB5 (grey bars) or 4LB5-HP-RNase (white bars). Measurements were performed at tumor excision.

To determine the therapeutic potential of 4LB5-HP-RNase *in vivo*, orthotopic mouse models of human TNBC were generated by injecting surface-NCL-positive MDA-MB-231 TNBC cells into the mammary fat pad of non obese diabetic-severe combined immunodeficient (NOD-SCID) mice. Two weeks post-injection of tumor cells, mice were divided into tumor-weight-graded groups, and injected i.p. with PBS solution or 2 mg/kg 4LB5-HP-RNase, twice a week. Four weeks after the first treatment, tumor volume was much smaller in 4LB5-HP-RNase-treated mice than in control mice (Figure 7A), thus indicating that 4LB5-HP-RNase exerts specific antitumor activity *in vivo*. Compared with control mice, 4LB5-HP-RNase treatment significantly inhibited tumor growth (*P* = 0.0098).

To compare the *in vivo* antitumor effects of 4LB5-HP-RNase with those observed for the parental 4LB5 scFv [24], we performed a second *in vivo* experiment in the same experimental model. Considering that the higher molecular weight of IR, compared to that of the parental scFv, could affect its permeation into large tumor masses, mice were used bearing tumors of reduced size that were injected with equimolar doses of 4LB5 or 4LB5-HP-RNase [24]. As shown in Figure 7B, the antitumor activity of 4LB5-HP-RNase was significantly stronger than that of 4LB5 (*P* = 0,0017) as well as a more significant reduction of tumor size (a third of control set; *P* = 0,0005) was observed in 4LB5-HP-RNase treated mice in comparison with that observed (half of control set) in mice bearing larger tumors. Interestingly, H&E staining shows several areas of reduced cellularity, potentially compatible with either activated tumor apoptosis or necrosis, after treatment with 4LB5-HP-RNase (Figure 7C, upper panels). Similar tissue alterations were also observed following 4LB5 treatment, in agreement with our previously published data [24]. Furthermore, Ki-67 staining of treated tumors (5–15% Ki67^+^ nuclei) indicates a reduced proliferation rate compared with untreated controls (60% Ki67^+^ nuclei). Notably, 4LB5 treatment resulted in a higher cytostatic effect (slightly lower value of Ki67^+^ nuclei) compared to that of 4LB5-HP-RNase, which is potentially compatible with increased cytotoxic properties conferred by the HP-RNase moiety to 4LB5. At the end of treatment, mice did not show significant signs of wasting, such as increased loss of weight (Figure 7D) or other detectable signs of toxicity, in comparison with control-treated mice, thus suggesting that neither 4LB5-HP-RNase nor 4LB5 exerts toxic effects on normal tissues.

## DISCUSSION

Despite the development of several targeted therapies (monoclonal antibodies, tyrosine-kinase and aromatase inhibitors) devised to transform breast cancer into a chronic disease, many patients show primary or acquired resistance to these anti-neoplastic drugs [39]. Indeed, unlike other types of breast cancer, for which targeted biological therapies are available, no targeted agent is yet available for TNBC. Consequently, new strategies for TNBC are urgently needed.

A new target for breast cancer (including TNBC) therapy is represented by Nucleolin (NCL). Nucleolin is a highly conserved nucleocytoplasmic protein that exerts many functional roles, localized in the nucleolus, nucleus and cytoplasm of the cell, which participates in many cellular functions under both physiological and pathological conditions. Recent reports show that NCL is frequently up-regulated and selectively expressed on the membrane cancer cells and cancer associated endothelial cells, but not on normal cells [10, 12–14].

Nucleolin is also a member of the rRNA nucleolar-processing complex [10, 40] and is directly involved in miRNA biogenesis [41], including that of miR-10a, miR-21, miR-103, miR-221 and miR-222. Since miR-21, miR-221 and miR-222 up-regulation has been associated with breast cancer aggressiveness and resistance to antineoplastic therapies [16–19], NCL exerts a critical pro-tumorigenic function by regulating their biogenesis at the post-transcriptional level, thus enhancing their maturation from pri- to pre-miRNAs [11].

Interestingly, miRNAs can also be detected in extracellular compartments [42–45] where they are stably “protected” because they are associated with RNA-binding proteins and lipoproteins or they can be incorporated into EVs [42, 46]. Furthermore, evidence of transfer of miRNAs from the bound proteins to the exosomes (and vice versa) [34], suggests a novel mechanism of intercellular communication in which RNA-binding proteins, such as NCL, may associate in complexes for the packaging and export of these miRNAs, thereby protecting them from protease degradation.

Given the continuous abundant expression of NCL on the tumor surface, inhibitors of surface NCL preferentially target tumor cells without affecting the nuclear NCL of normal cells [10]. We previously selected by phage display technology a fully human anti-NCL scFv, called “4LB5”, specifically directed against NCL on the cell membrane of cancer cells. This scFv selectively binds to NCL-positive tumor cells, inhibits their growth, and induces apoptosis. Furthermore, 4LB5 inhibits the production of mature miR-21, -221, and -222, by interfering in the NCL interaction with the microRNA microprocessor complex and impairing the maturation of precursors of these miRNAs [24].

Based on the ability of 4LB5 to translocate into the cytoplasm after binding to NCL, this scFv can be used to vehicle antitumoral molecules directly into cancer cells and so enhance their therapeutic activity. To evaluate this possibility, we engineered a novel anti-NCL immunoRNase, called “4LB5-HP-RNase”, by fusing the human scFv moiety with the human pancreatic RNase (HP-RNase), thus combining the antitumoral activity of 4LB5 with the 4LB5-HP-RNase enzymatic activity of the ribonuclease, which can be useful to degrade NCL associated miRNAs or intracellular RNAs.

Here we describe the construction, characterization and antitumor activity of the novel NCL-targeting immunoRNase. We demonstrate that 4LB5-HP-RNase retains the biological properties of both scFv and RNase. In fact, the novel immunoRNase selectively and efficiently binds to a panel of different surface-NCL-positive breast cancer cells but not to normal cells, and shows an enzymatic activity similar to that of the previously characterized IR (Erb-HP-RNase) [25]. Moreover, treatment with 4LB5-HP-RNase of breast cancer cells, but not of normal cells, significantly reduces their *in vitro* and *in vivo* growth by inducing both apoptosis and necrosis. Notably, the effect of 4LB5-HP-RNase on intracellular levels of tumorigenic miR-21, -221, and -222 in breast cancer cells is greater than that of 4LB5, which indicates that the acquired enzymatic activity improves the antitumoral efficacy of 4LB5 due to its ability to be internalized by nucleolin, as shown by both cytosolic and nucleolar localization of the immunoRNase (see Figure 3C), which is in line with the natural distribution pathway of NCL [23].

Finally, we report preliminary data suggesting that NCL plays a key role in EV-mediated cancer intracellular communication. In fact, we show for the first time that NCL is present in the exosomes released from cancer cells and that treatment with the anti-NCL immunoRNase greatly reduces EV-associated miR-21 levels. These data support the hypothesis that NCL is directly involved in EV-mediated intracellular communication and that molecules targeting NCL might be a means to impair this communication. This concept can be verified in future studies on the traffic of NCL and miRNAs in EVs and their molecular association. Another possible approach to test if the critical role of nucleolin is more involved in the extracellular exosomes or in the intracellular compartment could be represented by the construction of a novel immunoRNase made up of 4LB5 and a variant of HP-RNase made resistant to the intracellular Ribonuclease Inhibitor (RI), a cytosolic protein with a high affinity for HP-RNase [47, 48]. In this mutant protein, five amino acids of the ribonuclease at the RI–HP-RNase interface are replaced (R39D/N67D/N88A/G89D/R91D) in order to reduce the affinity for RI but still retaining high RNA-degrading activity [49]. If the efficacy of the variant immunoRNase is increased by the resistance to endogenous RI inhibition and the consequent antitumor activity becomes more efficient than that of the parental immunoRNase, described here, it will be possible not only to shed light on the mechanism of action but also to obtain a more potent immunoconjugate targeted to nucleolin in the future.

In summary, here we describe a new approach for the immunotherapy of TNBC based on a novel fully human NCL-targeting immunoconjugate that is endowed with potent antineoplastic activity. This treatment could be beneficial in types of carcinoma ineligible for currently available targeted therapies.

## MATERIALS AND METHODS

### Cell cultures

MDA-MB-436, BT549 and SW-620 cells were cultured in RPMI 1640 (Gibco BRL, Life Technologies, Paisley, UK); MDA-MB-231 cells were grown in Dulbecco's modified Eagle's medium (Gibco BRL); MCF-7 cells were grown in Eagle's Minimum Essential Medium (Gibco BRL). All media were supplemented with 10% (vol/vol) heat-inactivated fetal bovine serum (7.5% for JIMT-1), penicillin (100 UI ml^−1^), streptomycin (100 μg ml^−1^) and 2 mM glutamine (all from Gibco BRL). MCF-10a cells were cultured in Mammary Epithelial Cell Growth Medium (Lonza, Basel, CH) supplemented with 10% FBS, bovine pituitary extract, hydrocortisone, hEGF, insulin (Bullet Kit, Lonza) and 100 ng/ml cholera toxin. All cell lines were purchased from the American Type Culture Collection and cultured in a humidified atmosphere in 5% CO_2_ at 37°C.

### Antibodies

The following antibodies were used: horseradish peroxidase (HRP)-conjugated anti-penta-His mouse monoclonal antibody (Qiagen GmnH, Hilden, Germany), anti-NCL mouse monoclonal antibody (Santa Cruz Biotechnology Inc., Dallas, TX, USA), anti-PARP rabbit polyclonal antibody (Cell Signaling Technology, Danvers, MA, USA), anti-actin rabbit polyclonal antibody (Sigma-Aldrich, St Louis, MO, USA), HRP-conjugated anti-rabbit immunoglobulins from goat antiserum, HRP-conjugated anti-mouse immunoglobulins from goat antiserum (Thermo Scientific, Rockford, IL, USA).

### Construction, expression and purification of 4LB5–HP-RNase

The cDNA encoding the human pancreatic RNase was cloned into the T7 promoter-based *E. coli* pET22b+ expression vector downstream to the sequence encoding the available human 4LB5 scFv, followed by a spacer included to minimize the steric hindrance between the two moieties of the chimeric protein, as previously described [47]. The recombinant plasmid was fully sequenced to confirm the expected DNA sequence.

Cultures of Escherichia coli BL21 DE3 (Novagen, Merk Millipore, Darmstadt, Germany), previously transformed with the recombinant pET22b+ expression vector containing the cDNA of 4LB5-HP-RNase, were grown at 37°C in Luria–Bertani (LB) medium containing 50 μg/ml ampicillin until the exponential phase was reached. The expression of IR was induced by addition of 1 mM isopropyl-β-d-thiogalactopyranoside (IPTG; Applichem GmbH, Darmstadt, Germany) in the cell culture, which then was grown at room temperature for 4 h. The cells were harvested by centrifugation at 6,000 rpm for 15 min at 4°C. Recombinant protein was extracted from the insoluble fraction of pET22b(+)-4LB5-HP-RNase-transformed BL21 (DE3) bacterial cells. To this end, the fraction was solubilized in a PBS buffer containing 7 M UREA and incubated by slowly rotating overnight at 4°C in the presence of EDTA-free protease inhibitors (Roche Applied Science GmbH, Manheim, Germany). After centrifugation at 28,000 g for 20 min at 4°C the supernatant was incubated with a 100% saturated solution (4 M) of ammonium sulfate, added drop-wise at 4°C, to allow protein precipitation. The mixture was incubated for 1 hour at 4°C under slow stirring and then centrifuged at 28,000 g for 10 min at 4°C. The precipitated material was resolubilized in resin binding buffer (20 mM sodium phosphate, 0.5 M NaCl, 10 mM imidazole pH 7.4) in the presence of 4 M UREA and EDTA-free protease inhibitors, and incubated for a further 18 h at 4°C under slow stirring. After centrifugation at 28,000 g for 10 min at 4°C to remove unsoluble proteins, the cleared supernatant was loaded on an immobilized-metal affinity chromatography (IMAC) by incubation with cobalt-chelating resin (TALON^®^; Clontech, Palo Alto, CA, USA) overnight at 4°C under gentle rotation. After extensive washes in an appropriate buffer (20 mM sodium phosphate, 0.5 M NaCl, 20 mM imidazole) by decreasing urea concentration in 10% glycerol (vol/vol), the elution step was performed with the same buffer containing a higher concentration of imidazole (250 mM). The purified protein was analyzed by RNase zymograms, carried out by SDS-PAGE electropherograms as previously described [50]. Briefly, the gel was incubated in a buffer of 50 mM Tris-HCl pH 7.4 containing 25% isopropanol for 30 minutes to increase the sensitivity of the signal detection. The gel was equilibrated in a buffer of 50 mM Tris-HCl pH 7.4 for 30 minutes and then incubated in the same solution containing 4 mg/ml yeast RNA (Sigma-Aldrich) at 37°C for 10 min. The gel was washed out with a buffer of 50 mM Tris-HCl pH 7.4 and stained in a solution of 0,2% toluidine blu containing 0,1% acetic acid solution for 15 minutes at room temperature by gentle agitation. Finally, the gel was destained by extensive washes in water. Erb-HP-RNase was used as positive control in a parallel assay.

### ELISA assays

Binding assays were performed by modified enzyme-linked immunosorbent assay (ELISA), as previously described [25]. ELISA assays for 4LB5-HP-RNase or 4LB5 were performed on surface NCL-positive MDA-MB-231, MDA-MB-436, BT549 or MCF-7 breast cancer cells and surface NCL-negative MCF10a normal-like breast cells. Briefly, all the cells were harvested in non-enzymatic dissociation solution (Sigma-Aldrich), washed and transferred to U-bottom microtiter plates (2 × 10^5^ cells per well). After blocking with PBS containing 6% bovine serum albumin (BSA), the cells were incubated in the absence or in the presence of increasing concentrations of IR in ELISA buffer (PBS/BSA 3%) for 90 min at 25°C by gentle shaking. After centrifugation at 1200 rpm for 7 min and the removal of supernatant, cell pellets were washed twice in 200 μl of PBS buffer, resuspended in 100 μl of ELISA buffer and incubated for 1 h with a peroxidase-conjugated anti-Histidine tag antibody. After 1 h, the plates were centrifuged, washed with PBS buffer and reacted with 3,3′,5,5′-tetramethylbenzidine (Sigma-Aldrich). The enzymatic reaction was stopped with 1 N HCl, and the binding values were determined from the absorbance at 450 nm and reported as the mean of three determinations. The SD (≤ 5%) was calculated on the basis of the results obtained in three independent experiments.

To further confirm that the observed binding was due to the specific interaction between 4LB5 and NCL on cell surface, the binding of 4LB5-HP-RNase to MDA-MB-231 cells was analyzed by ELISA assay before and after its pre-incubation with equimolar doses (50 nM) of NCL-RBD (RNA Binding Domain) recombinant protein. To this end IR was pre-incubated for 2 hours at 4°C with NCL-RBD recombinant protein by gentle rotation and then tested on MDA-MB-231 cells as described above.

### Pull-down assay

Subconfluent MDA-MB-231 cells were harvested and lysed in NETN lysis buffer (150 mM NaCl, 0.2 mM EDTA, 50 mM Tris-HCl at pH 7.5, 1% [vol/vol] NP-40) containing protease inhibitors. The BCA Protein Assay kit was used to measure the concentration of the whole cell extracts (WCEs). An aliquot of 2 mg of total protein extract was incubated with equimolar doses (100 nM) of 4LB5 or 4LB5-HP-RNase, or with control buffer (PBS) and loaded on Ni-NTA Magnetic Agarose Beads for 2 h at room temperature under continuous stirring to enable capture of the chimeric proteins. The resin was washed out four times with 1 ml of NETN lysis buffer containing 0.25% NP-40. The samples were boiled in 2× SDS sample buffer, run on SDS-PAGE gel, transferred to PVDF and probed with appropriate dilutions of anti-NCL or anti-His antibodies.

### *In vitro* cytotoxicity assays

MDA-MB-231 and MDA-MB-436 cells were plated in 96-well plates at a density of 7 × 10^3^ per well; BT-549, MCF-7 and MCF10a cells at density of 1 × 10^4^ per well. Cells were treated with the increasing concentrations (10–100 nM) of 4LB5-HP-RNase and incubated at 37°C for 72 h. Cells counts were obtained with the Trypan blue exclusion test or by MTT assay. For MTT assays, cells were seeded in 96-well plates in complete growth media and after 24 h, cell culture media were replaced with fresh media in the absence or in the presence of 4LB5-HP-RNase. After 72 h aliquots of MTT (Sigma-Aldrich) were added to each well and plates were incubated at 37°C for 2–3 h. The reaction was monitored at A490 nm.

Cell survival is reported as percentage of viable cells in the presence of the protein under test versus untreated cells. Typically, cell survival values were obtained from at least three independent experiments in which five determinations were performed for each sample. Standard deviations were calculated on the basis of the results obtained from all the experiments.

### Colony assays

MDA-MB-231, MDA-MB-436 or BT-549 cells (200 cells/well) were plated in 6-well plates and treated with 100 nM 4LB5-HP-RNase or 4LB5 in complete medium for 72 h. Then, cells were incubated with complete medium without proteins and allowed to grow for an additional 7 days to obtain the colonies. Cells were then fixed with 1% glutaraldehyde in PBS, stained with Crystal violet and counted.

### Purification of EVs

SW620 cells at 80–90% confluency were washed twice with PBS and then incubated in serum-free RPMI-1640 for 24 h to prevent potential contamination from serum-derived vesicles and proteins. The conditioned medium was collected and cells/cell debris was removed from the cell culture supernatant with a combination of differential centrifugation, as follows: 50 g for 20 min, 100 g for 20 min, 300 g for 10 min, 2000 g for 20 min and 2000 g for 45 min. The cell culture supernatant was recovered and further clarified by filtration through a 0.22 μm filter (Millipore). EVs were isolated by adding ExoQuick^™^ exosome precipitation reagent according to the manufacturer's recommendations and incubated overnight at 4°C to enable EV precipitation. The ExoQuick/supernatant mixture was centrifuged at 1500 g for 30 min and the supernatant was removed. The EV pellet was resuspended in a sterile nuclease-free PBS solution and incubated with equimolar doses of 4LB5 or 4LB5-HP-RNase (50 nM) at 37°C for 2 h or with a PBS control solution.

### RNA extraction and quantitative real-time PCR

RNA was purified with the “RNA clean-up and concentration” micro kit (Norgen) according to the manufacturer's instructions, after phenol/guanidine-based extraction. For RNA extraction from EVs, Ath-miR159a and cel-miR-248 synthetic oligos were added to each sample to normalize the results of quantitative RT-PCR. Quantitative real-time PCRs were performed with the TaqMan Fast-PCR kit (Applied Biosystems) according to the manufacturer's instructions, using the appropriate TaqMan probes for miRNA quantification, followed by detection with the 7900HT Sequence Detection System (Applied Biosystems). All reactions were performed in triplicate. Simultaneous quantification of GAPDH was used as reference for intracellular miRNA quantification. Simultaneous quantification of RNU48 was used as reference for exosome-related miRNA quantification. The comparative cycle threshold (Ct) method for relative quantification of gene and miRNA expression (User Bulletin #2; Applied Biosystems) was used to determine miRNA expression levels.

### *In vivo* antitumor assays

To test the *in vivo* activity of 4LB5-HP-RNase, 2 × 10^6^ viable Luc+ MDA-MB-231 cells were injected into the fourth left-side mammary fat pad of female NOD-SCID mice (NOD/ShiLtSz; Charles River). Two weeks after tumor cell inoculation, mice were treated twice a week with i.p. injections of 4LB5, 4LB5-HP-RNase (2 mg/kg each) or control buffer (PBS). After 4 weeks of treatment, mice were euthanized and tumors excised. Tumor size was measured with a caliper, and the volume was calculated with the formula L × W × H. Tissue preparation and staining were performed as previously reported [24]. At least 10 different fields containing at least 100 cells each were analyzed and counted for the relative abundance of Ki67 positive and negative nuclei.

### Internalization experiments

Internalization of 4LB5-HP-RNase was assessed by confocal microscopy. 4LB5 and 4LB5-HP-RNase were Cy5-labeled using the LYNX Rapid Cy5 antibody conjugation kit (AbD Serotec), according to the manufacturer instructions. Sub-confluent MDA-MB-231 cells were seeded over a glass multi-well, treated with 1 μg/mL of Cy5-4LB5 or Cy5-4LB5-HP-RNase diluted in complete medium and cultured at 37°C for 4 h to enable their internalization. Cells were extensively washed with PBS, and complete medium (without Cy5-labeled antibodies) was replaced, after the addition of Hoechst dye for nuclear staining. Live cell imaging was performed by using an A1 plus laser-scanning, confocal microscope equipped with a 60× oil objective lens (N.A. 1.4) (Nikon). All imaging for the detection of Cy5 was performed by using the same instrument settings. Images were processed for presentation using NIS-Elements software (Nikon). Imaris8 (version 8.2.0) was used to determine the Cy5 mean fluorescent intensity and intracellular and extracellular signals were segmented by using the DIC images.

### Statistical analysis

The values reported in the graphs were obtained from at least three independent experiments in which three determinations were performed for each sample, and standard deviations were calculated on the results of all the experiments. Results are expressed as the mean + 1 standard deviation of the mean. The differences between the mice groups in *in vivo* studies were analyzed with unpaired two-tailed Student's *t* test. *P* < 0.05 was considerated statistically significant and indicated with asterisks as described in the Figure legends.

### Study approval

Animal experiments were performed according to the Ohio State University institutional guidelines after review by the institutional review board.

## SUPPLEMENTARY VIDEO




