# Supplementary material for: A novel fully human anti-NCL immunoRNase for triple-negative breast cancer therapy
Source: Oncotarget. 2016 Nov 23;7(52):87016–30. doi: 10.18632/oncotarget.13522 (PMC5349967; doi:10.18632/oncotarget.13522)
Supplement: Supplementary file 1 [file oncotarget-07-87016-s001.pdf]

A novel fully human anti-NCL immunoRNase for triple-negative breast cancer therapy

Supplementary Materials

Supplementary Video S1: Tridimensional rendering of 4LB5-HP-RNase internalization in breast cancer cells.
